# From the threat to the large outbreak: dengue on Reunion Island, 2015 to 2018

**DOI:** 10.2807/1560-7917.ES.2019.24.47.1900346

**Published:** 2019-11-21

**Authors:** Muriel Vincent, Sophie Larrieu, Pascal Vilain, Aurélie Etienne, Jean-Louis Solet, Claire François, Bénédicte Roquebert, Marie-Christine Jaffar Bandjee, Laurent Filleul, Luce Menudier

**Affiliations:** 1 Santé Publique France, Saint Denis, France; 2 Centre Hospitalier de l’Ouest Réunion, Saint Paul, France; 3 Centre Hospitalier Universitaire de la Réunion, Saint Denis, France

**Keywords:** Dengue, Indian Ocean, Resurgence, Surveillance, Outbreak, dengue virus type 2

## Abstract

**Background:**

With more than 300 million infections estimated annually worldwide, dengue is the most prevalent arboviral infection. On Reunion Island, after a large outbreak in 1977–78, only limited episodes of viral circulation or sporadic cases were reported till 2015.

**Aim:**

Our objective was to document and report on the circulation of dengue virus after the occurrence of a small outbreak during austral summer 2015/16 and until the large outbreak of 2018.

**Methods:**

Beside the mandatory notification of biologically confirmed dengue cases, additional systems of surveillance were set up: estimation of dengue-like syndrome in people seeking care by their family doctor, surveillance of emergency department visits related to dengue, surveillance of hospitalised dengue patients and deaths classifications.

**Results:**

After a moderate outbreak during summer 2015/16 with 231 cases, 2017 was characterised by limited viral circulation (97 cases) which, however, persisted during the austral winter. By February 2018, the number of cases had increased and led to a peak at the beginning of May 2018. More than 6,000 cases were reported this year (dengue virus type 2 only). In addition, six deaths of dengue patients were notified.

**Conclusion:**

In 2017, the persistence of transmission during winter created favourable conditions for the emergence of an epidemic during summer 2018. After this moderate epidemic wave, the viral circulation persisted during winter 2018 for the second year, opening the door for the second wave in 2019 and for potential endemisation of the disease on Reunion Island in the near future.

## Background

Once restricted to South East Asia and probably under-reported, dengue progressively became the most prevalent arboviral disease. Caused by four viral serotypes (DENV-1, DENV-2, DENV-3 and DENV-4), dengue is transmitted by *Aedes aegypti* and *Aedes albopictus* mosquitoes. Transmission is now reported in at least 128 countries and almost 4 billion people are at risk worldwide [1,2]. In 2013, Bhatt et al. estimated that the number of dengue virus infections per year reached 390 million among which 96 million were symptomatic [3]. Dengue is influenced by many factors including environment, population density and climatic conditions. The (re)emergence of the disease is probably largely due to the combination of urbanisation, climate changes and globalisation [4]. Mosquitoes, major determinants of arbovirus occurrence and dispersion, have the ability to adapt to the increasing urbanisation and the land perturbations. As a consequence, their population increases together with their settlement areas [5,6].

Dengue is an acute systemic disease characterised by a range of clinical expressions [3,7,8]. Some estimates reach up to 75% of asymptomatic or, more precisely, paucisymptomatic forms [3,9]. Nonetheless, it is estimated that ca 500,000 people are hospitalised for a severe dengue episode each year and the case fatality rate reaches 2.5% [7]. Infection provides lifelong immunity against the same serotype but only short-term protection against heterogeneous serotypes. Secondary infection with another serotype raises the risk to develop a severe dengue episode associated with increased morbidity and mortality [10]. The increasing dengue prevalence combined with its geographical extension are therefore a public health threat.

There is no specific treatment, and prevention relies on individual protection against mosquito bites and on vector control measures. A dengue vaccine (Dengvaxia) has been developed. French authorities only recommend it after individual screening for past infection with highly specific tests [11,12]. If this is not feasible, vaccination should only be considered in areas where the seroprevalence in people older than 9 years is higher than 80%. Also, the vaccine should not be used in non-endemic areas in the context of an epidemic. Vaccination is therefore not recommended for Reunion Island [12,13].

Reunion Island is a French overseas territory located in the south-western Indian Ocean (southern hemisphere). Its subtropical climate with mild winters and warm summers is suitable for the development of *Aedes* mosquitoes, and throughout the year (whatever the season), Breteau indexes (a measure for the density of mosquitoes in an area [14]) are compatible with the persistence of viral circulation and therefore a potential epidemic start. Since the 1950s, *Ae. albopictus*, the vector of dengue viruses (DENV-1–4), has been the dominant species in this territory [15]. In 1977/78, a large epidemic occurred on the island, with an estimated 30% of the population infected [16]. Afterwards and until 2015, only sporadic autochthonous cases of dengue virus were confirmed (3–31 cases each year) and a limited circulation episode was described in 2004 (228 cases) [17,18]. 

The aim of this paper was to describe the findings from dengue surveillance between November 2015 and the end of December 2018 and to document how surveillance systems were adapted to better reflect the dynamic of the epidemic. The ongoing epidemic of 2019 is not presented here.

## Methods

### Case definitions

Dengue-like syndrome was defined as acute fever associated with one or more of the following signs or symptoms: nausea, vomiting, rash, headache, retro-orbital pain, myalgia, arthralgia or haemorrhagic signs. A probable case was a case with dengue-like syndrome in whom IgM antibodies against dengue virus were detected and who had epidemiological, biological and/or clinical arguments in favour of a dengue infection (link to a confirmed case, recent travel in a dengue-endemic area or an active viral circulation zone, marked dengue-like syndrome with IgM). A confirmed case was a person with biological confirmation of DENV infection, i.e. RT-PCR, seroconversion (fourfold increase in IgG titre between two samples taken 2 weeks apart) or positive seroneutralisation assay. Imported and autochthonous cases were, respectively, cases with and without history of travel to a dengue-endemic area within the 15 days before symptoms onset. In the Results section, autochthonous cases refers to the total of probable and confirmed cases.

### Laboratory confirmation

Detection of the viral genome by RT-PCR is the gold standard and was performed for every sample collected within 5 days after the onset of symptoms. Between day 5 and day 7, both RT-PCR and serological analysis were performed, while after day 7, only serological testing is relevant.

### Epidemiological surveillance

On Reunion Island, epidemiological surveillance of arboviral diseases has been undertaken since 2004 by the regional unit of the French national public health agency (Santé publique France– La Réunion) in collaboration with the French Indian Ocean health agency (ARS OI). In inter-epidemic periods, the surveillance of dengue relies on the national mandatory reporting, supplemented by the transmission of all positive results by all laboratories doing biomedical analyses. Biological analyses are performed on a clinician’s request for any patient with dengue-like syndrome. Each case is documented and classified according to the specific case definitions. As soon as a case is notified, control measures are implemented by the vector control team, Lutte antivectorielle (LAV) of the ARS OI and active case finding among the patient’s contacts is performed.

With the onset of an epidemic, additional surveillance systems were progressively set up. Since 2016, patients with a dengue diagnosis and hospitalised for more than 24 hours have been reported to Santé Publique France – La Réunion by clinicians on a voluntary basis. As recommended by the World Health Organization (WHO) and described in the International Classification of Diseases [19], warning signs (abdominal pain or tenderness, mucosal bleeding, lethargy and/or restlessness, rapid decrease in platelet count, increase in haematocrit, persistent vomiting, visible fluid accumulation or liver enlargement) and signs of severity (severe plasma leakage leading to shock and/or fluid accumulation with respiratory distress, severe bleeding or severe organ impairment) are monitored. This surveillance, which provides key indicators of the severity of an outbreak, has been extensively described [20].

In addition, since February 2018, the weekly number of visits to each of the six emergency departments (EDs) of Reunion Island that are related to dengue-like illness have been reported to Santé Publique France – La Réunion through an automated system monitoring the activity of EDs (OSCOUR network, Organisation de la Surveillance Coordonnée des Urgences) [21].

Since May 2018, a dedicated committee has investigated deaths of patients with a confirmed or a probable diagnosis of dengue. Deaths are classified as directly linked, indirectly linked or unrelated to dengue infection based on an algorithm developed in the French West Indies [22].

Lastly, in the beginning of 2018, the sentinel physicians’ network (52 general practitioners and two paediatricians), which monitors and weekly reports surveillance data for several pathologies and syndromes to Santé Publique France – La Réunion [23], was requested to participate in the dengue surveillance. Sentinel physicians report the number of dengue-like syndromes seen during their consultations. Based on the total number of medical consultations performed each week on Reunion Island (data transmitted by the national health insurance), the total number of dengue-like syndromes in people seeking care is extrapolated. It provides additional reliable information on the dynamic of the epidemic [21,24,25].

### Ethical statement

As an epidemiological record, no ethical approval was needed. Each dengue case is reported to the regional health agency (ARS OI) to conduct vector control measures. For the purpose of population’s health surveillance and support to decision making (some of the missions of Santé publique France), local health authorities must give access to the regional team of Santé publique France to health data related to each of the 33 mandatory diseases – dengue being one of them.

## Results

### Epidemiological and demographic description

#### Moderate seasonal circulation during summer 2015/16

**Figure 1:**
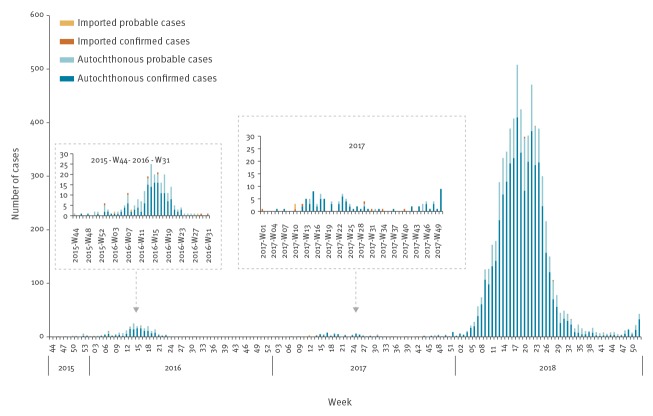
Epidemic curves of autochthonous and imported dengue cases reported by week of symptom onset, Reunion Island, November 2015–December 2018 (n = 7,127)

The first cases were detected in November 2015. The viral circulation started to increase in March and peaked in April (week 14) with a total of 25 reported cases (14 confirmed and 11 probable) (Figure 1). The number of reported cases started to decrease in June and the last autochthonous case was reported in July.

Between November 2015 and August 2016, 240 cases of dengue were identified: 231 were acquired locally while nine were imported by travellers returning from Indonesia, Malaysia, Nicaragua, the Seychelles or Thailand. The male/female sex ratio was of 0.9 and the median age of cases was 38 years (range: 2–92 years).

During this episode, three serotypes were identified: 69 DENV-1, 17 DENV-2 and 10 DENV-3 (Table 1), probably linked to introduction by travellers returning from endemic areas. Among the nine imported cases, three serotypes were identified by serotyping in three travellers returned from Indonesia and Malaysia. Epidemiological investigations showed links between imported and autochthonous cases of the same serotype suggesting the existence of at least three different transmission chains during this outbreak.

**Table 1:** Proportion of each dengue virus serotype per year, among autochthonous serotyped cases, Reunion Island, November 2015–December 2018 (n = 951)

Years	Total number of cases	Total of serotyped cases	DENV1		DENV2		DENV3		DENV4	
			n	%	n	%	n	%	n	%
2015/16	231	93	68	73.1	16	17.2	9	9.7	0	0
2017	97	56	2	3.6	53	94.6	0	0	1	1.8
2018	6,770	951	0	0	951	100	0	0	0	0

#### Continuous low-level transmission in 2017

In 2017, the first autochthonous case was detected in February. The number of reported cases then slowly increased until April (8 cases in week 17), plateaued with the beginning of winter and then decreased. While the average number of cases detected every week remained low (less than 10 cases per week), the transmission persisted throughout the year (Figure 1). In total, 106 dengue cases were declared on Reunion Island in 2017 and among them, nine were imported (from India, Indonesia, Myanmar, the Seychelles, Sri Lanka and Thailand). The male/female sex ratio was 1.1 and the median age was 47 years (range: 6–81 years).

Serotyping was performed for 61 samples of which five were from imported cases. DENV-2 was the most frequent, both in autochthonous and in imported cases. Of note, DENV-4 was isolated in an autochthonous case (Table 1).

#### The 2018 outbreak

In 2018, from two cases in week 2 (January), the weekly number of cases quickly escalated to 21 in week 5 (February) and 130 in week 9 (March). The outbreak then expanded and peaked in week 18 (May) with 495 cases. The active epidemic period ranged between week 13 (March) and week 27 (July). Thereafter, the number of cases plateaued until week 24 (June), started to decrease with the beginning of the austral winter and stabilised at ca 10 cases per week. The number of reported cases started to increase again by the end of December (Figure 1).

By the end of the year, 6,781 cases had been reported of which 11 were imported (from Brazil, Malaysia, the Maldives, Myanmar, Polynesia and Thailand). A total of 5,383 (78.9%) autochthonous cases were biologically confirmed. The male/female sex ratio was 1.1 and the median age was 42 years (range: 0–96 years).

During this outbreak, 951 samples were serotyped and in autochthonous cases, DENV-2 was the only serotype identified (Table 1). Serotyping was performed in all confirmed imported cases: DENV-1 was identified in four samples (travellers back from Brazil, Malaysia, Myanmar and Thailand) while DENV-2 and DENV3 were isolated in two samples each (travellers returned from India, the Maldives and Thailand).

Based on the weekly reports transmitted by the sentinel physician’s network and data from the National Health Insurance, it was estimated that between week 13 and week 27, more than 15,000 people affected by dengue-like syndrome consulted a doctor. During this plateau, the ratio between confirmed and probable cases vs estimates was almost stable, at ca 33% (range: 22–43%) (data not shown).

### Geographical distribution: 2015–2018

**Figure 2:**
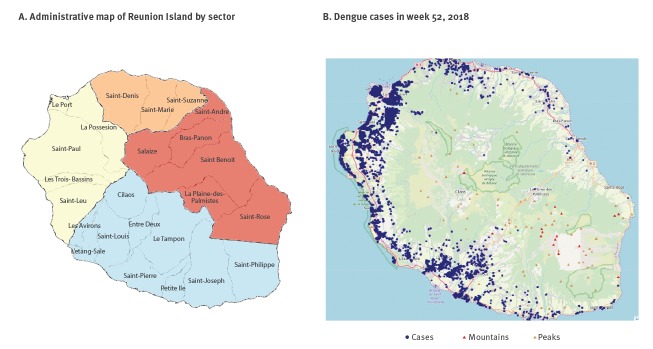
Geographical distribution of autochthonous and imported dengue cases, Reunion Island, 2018 (n = 6,781)

During the 2015/16 outbreak, more than 65% of all cases (autochthonous and imported) were located in the south, and ca 30% in the west (Figure 2A for sectors, and Table 2).

**Table 2:** Autochthonous dengue cases reported by region and per year, Reunion Island, November 2015–December 2018 (n = 7,098)

Years	Total number of cases	North		East		South		West		Missing addresses
		n	%	n	%	n	%	n	%	
2015/16	231	8	3.5	2	0.8	152	65.8	69	29.9	0
2017	97	2	2.1	3	3.1	35	36.1	57	58.8	0
2018	6,770	213	3.1	39	0.6	1,304	19.3	5,183	76.6	31

In 2015/16, persistent viral circulation was observed in five municipalities, four of them located in the south and one in the west. The northern and eastern parts of the island were barely affected, reporting eight and two cases, respectively. Imported cases followed the same distribution. In 2017, the situation changed and almost 60% of the cases were declared in the densely populated western region, while ca 35% of the cases were located in the south (Table 2). In the west, more than 40% of all cases (autochthonous and imported) were declared in only one municipality. Only one focus of active viral circulation was identified in the south. Again, northern and eastern parts of the island were barely affected. After the low-grade, but persistent, viral transmission during the austral winter of 2017, the first cases reported in 2018 occurred in the areas with active foci in 2017. However, from a situation that initially was geographically and numerically limited, the number of cases quickly escalated and a wide geographical dispersion was observed even if mostly located in the west. By the end of summer, all municipalities had reported at least one case except for one municipality that was isolated and located ca 1,000 m above the sea level (Figure 2B). Of note, the distribution of cases remained heterogeneous in the affected areas and while the reporting rate almost reached 10% in some neighbourhoods (data not shown), some others remained barely affected.

### Clinical expression of the disease

**Figure 3:**
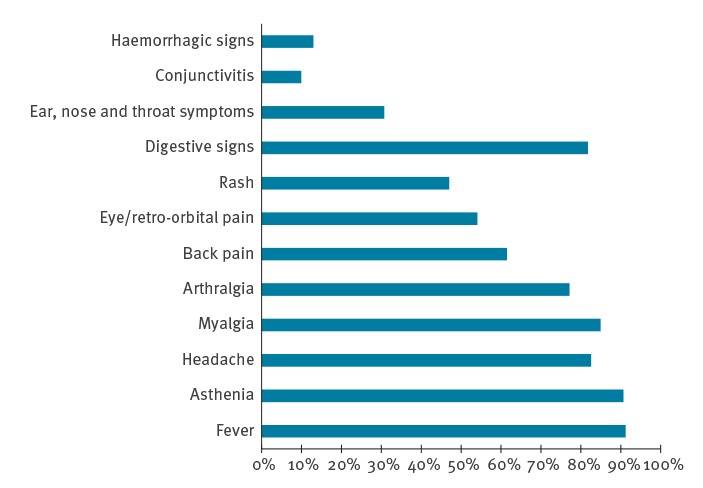
Clinical signs and symptoms among dengue cases, Reunion Island, November 2015–December 2018 (n = 3,958)

The clinical signs that were consistently the most common in each year of the studied period were fever, followed by symptoms such as asthenia, headache, myalgia, arthralgia, back pain and eye/retro-orbital pain. The cumulative frequency throughout the whole study period is shown in Figure 3. Digestive signs, cutaneous rash, ear, nose and throat symptoms, conjunctivitis or haemorrhagic signs were present at variable proportions between years (not shown). Of note, in 2018, clinical signs could only by collected for 53% of patients owing to the heavy workload caused by the outbreak.

During the 2015/16 outbreak, 18 patients (7.5%) required hospitalisation because of warning signs as defined by the WHO and three experienced a severe form of the disease. In 2017, 14 patients (13%) were hospitalised because of warning signs. In 2018, 156 patients (2.3%) were hospitalised and among them, 27 suffered from severe dengue. A summary of this population is presented in Table 3. The male to female sex ratio was 1.1 for cases versus 0.86 for hospitalised patients. The percentage of patients 65 years and older was higher among hospitalised cases.

**Table 3:** Description of autochthonous dengue cases and hospitalised dengue patients, Reunion Island, 2018 (n = 6,770)

	Autochthonous cases		Hospitalised	
	n	%	n	%
Number of cases (male/female)	6,770 (3,497/3,273)		156 (72/84)	
Median age (range)	42 (0–96)		55 (0–88)	
0–14 years	555	8	7	4
15–64 years	5,334	79	95	61
≥ 65 years	866	13	54	35
Dengue without warning signs	Not recorded		41	29^a^
Dengue with warning signs	Not recorded		102	71^a^
Severe dengue	Not recorded		27	18^a^
Risk factors (n = 149)	Not recorded		44	29
*- Pregnancy*	Not recorded		*16*	*36*
*- Sickle cell anaemia*	Not recorded		*0*	*0*
*- Immunosuppression*	Not recorded		*9*	*20*
*- Platelet function disorder*	Not recorded		*1*	*2*
*- Other*	Not recorded		*17*	*39*
Median hospital length of stay (range)	Not applicable		4 (1–24)	

^a^ Clinical information only available for 149 of the 156 hospitalised cases.

Of note, one case occurred in a neonate from a mother diagnosed several days before delivery, strongly suggesting vertical transmission. Among hospitalised patients, thrombopenia and lethargy were the most common warning sign (ca 30%; data for the other signs not shown). Thrombopenia was present in half of the patients hospitalised for a severe dengue episode, followed by renal (n = 9) and hepatic (n = 7) failure (data for the other signs not shown).

Data from the OSCOUR network showed that in 2018, 475 people sought care at an ED for dengue-like syndrome and again, the western and southern regions were more affected. Hospitalisations and emergency consultations closely followed the temporal dynamic of the epidemic.

Between May and September 2018, six deaths were reported in patients with a confirmed or probable dengue diagnosis and a patient with dengue-like syndrome (possible case). After clinical investigations, three deaths were classified as directly linked to dengue infection and three as indirectly linked.

## Discussion

Since the large dengue epidemic that affected the island in 1977/78, only sporadic cases or minor epidemics had been reported on Reunion Island. After a moderate episode in 2016, the uninterrupted circulation throughout 2017 created a risk for a larger outbreak at the beginning of the following summer. By February 2018, it became apparent that an epidemic had started. Initially geographically limited to the west and south of Reunion Island, the outbreak spread progressively across the coastal band from the west to the south. The wide spread across a large area substantially hampered the vector control measures taken by a well-dimensioned and reactive department. By the end of the year, the number of reported autochthonous cases reached 6,770 cases and again, the transmission persisted during winter creating favourable conditions for another outbreak in 2019. In addition, the case distribution in 2018 was very heterogeneous, leaving an important part of the population, probably naïve [13], at risk. At the time of writing, the ongoing epidemic of 2019 had reached some of the previously unaffected areas and the case count had reached more than 18,000 autochthonous cases in the beginning of November (preliminary data). In the past, such a dynamic, i.e. low-level uninterrupted transmission throughout the year leading to a larger outbreak in the following summer, has already been described on the island: A large chikungunya epidemic in 2006/07 that affected more than 30% of the population had the same dynamic and was transmitted by the same vector (*Ae. albopictus*) [24]. The low level of immunity against dengue virus in the population of Reunion Island may explain this two-stage dynamic [13].

Early identification of all dengue cases by the surveillance system allows the immediate implementation of vector control measures in order to limit the spread of local transmission chains. However, a major difficulty regarding the management of a dengue epidemic is the large proportion of paucisymptomatic individuals (between 50 and 90% according to the setting) [3,26-28] which could contribute to transmission and spread of the disease but remain undetected by the surveillance system [10]. A better knowledge of asymptomatic cases therefore represents an important public health priority on Reunion Island and could be assessed by seroprevalence studies in asymptomatic relatives of confirmed cases.

During an inter-epidemic period, the surveillance and confirmation of any suspected case is of the outmost importance. Indeed, systematic confirmation can detect a potential resurgence of the epidemic and the emergence of new viral transmission areas. In contrast, during the course of an epidemic, population surveillance gives access to reliable and robust estimates of people affected by the disease and seeking care [21,23], as suggested by the stable ratio of confirmed vs estimated cases that we observed during the epidemic peak. As a consequence, these data obtained from the network of sentinel physicians are crucial to the proper surveillance of dengue.

Monitoring of hospitalised patients also provides essential indicators to assess the severity of the epidemic but is very time-consuming and not exhaustive (50% for 2018 [20]). Reflections are therefore currently ongoing to improve this part of the surveillance.

While infection with a DENV serotype confers lifelong protection against the same serotype, it not only gives a short protection against another serotype but increases the risk to develop a severe form of the disease during a second infection. While in 2018, DENV-2 was the only serotype detected, the risk is real for another serotype to get established on Reunion Island. Indeed, the island has multiple commercial, family and touristic exchanges with a large range of countries where the disease is endemic and where other serotypes circulates [29,30]. The co-circulation of several serotypes would create a risk for people previously infected by the DENV-2 serotype to develop a severe form of the disease if contracting a second infection. The monitoring of circulating serotypes therefore remains a priority for 2020. In addition, the surveillance of hospitalised patients should be able to detect an increase in severe forms.

Over the years, dengue on Reunion Island has progressively expanded from sporadic cases and limited small outbreaks to a large epidemic. This situation parallels the worldwide increase in the occurrence of this disease and the growing number of population exchanges with endemic areas (tourism, family visits, business trips, etc). Altogether, this creates suitable conditions for the introduction of the virus on Reunion Island, considering the low immunity of the population and a vector density that remains compatible with local viral transmission throughout the year. Despite links with mainland France and other countries in Europe where *Aedes* vectors are expanding, recent findings suggest that the risk of dengue getting established in Europe remains low [31,32]. Whether the disease will disappear from Reunion Island after the current wave in 2019 – as we saw after previous large outbreaks of dengue and chikungunya – or whether it will evolve to seasonal epidemics or to endemicity is unknown so far. Complete extinction of the circulation however seems highly unlikely. Surveillance findings coupled to post-epidemic seroprevalence surveys are crucial elements for appropriate policies regarding dengue control on the island.
